# Posttraumatic growth and depreciation six years after the 2004 tsunami

**DOI:** 10.1080/20008198.2017.1302691

**Published:** 2017-03-24

**Authors:** Hans Michélsen, Charlotte Therup-Svedenlöf, Magnus Backheden, Abbe Schulman

**Affiliations:** ^a^ Crisis and Disaster Psychology, Department of Neurobiology, Care Sciences and Society, Karolinska Institutet, Huddinge, Sweden; ^b^ Department of Learning, Informatics, Management and Ethics, LIME, Karolinska Institutet, Stockholm, Sweden

**Keywords:** Natural disaster, posttraumatic stress, psychological distress, posttraumatic growth, posttraumatic depreciation, longitudinal studies

## Abstract

**Background**: Posttraumatic growth (PTG) has been reported after various types of potentially traumatic events, as a part of the personal recovery process among survivors. Even negative changes in survivors’ life view, known as posttraumatic depreciation (PTD), have been identified as an additional aspect in the personal recovery processes.

**Objective**: To examine how the type of exposure experienced by survivors of a natural disaster, the 2004 Southeast Asia tsunami, influenced self-reported PTG and PTD six years later (T2). Additionally, the study examined the relations between psychological distress and posttraumatic stress symptoms (PTSS) 14 months after the disaster (T1), to PTG and PTD, respectively at T2. Finally, the study examined whether psychological distress and PTSS (T1) could have a mediating effect on PTG and PTD at T2.

**Method**: The participants were 848 tsunami survivors living in Stockholm, Sweden who responded to a questionnaire at 14 months (T1) and six years (T2) after the tsunami. The material was analysed using linear regression and pathway analysis. PTG and PTD were measured on separate scales.

**Results**: The type of exposure was significant related to both PTG and PTD six years later (T2). Those experiencing a combination of various types of exposure (including threat to life and bereavement) reported higher scores for both PTG and PTD. There were significant positive correlations between PTSS at T1 and PTG /PTD at T2, and somewhat lower correlations between psychological distress at T1 and PTG/PTD at T2. Both PTSS and psychological distress at T1 were significant mediating variables for both PTG and PTD at T2.

**Conclusions**: Studying survivors’ various types of exposure and subsequent changed view of life – both PTG and PTD – resulted in a broadened understanding of the complexity of reactions and the recovery process among survivors.

## 1. Introduction

Natural disasters, like other serious events, can be potentially traumatic for those afflicted, and many survivors initially show very strong psychological reactions. Most survivors recover (Galea, Nandi, & Vlahov, 2005; Norris et al., 2002), but for some, the disaster may lead to a variety of psychological problems such as posttraumatic stress disorder, depression, or anxiety (Neria, Nandi, & Galea, 2008; Shalev et al., 1998). In order for survivors to manage dire and often life-threatening events without developing serious psychological symptoms, they need various types of adaptive coping mechanisms (Bonanno, 2004). Many studies have noted that those afflicted by potentially traumatic events have eventually reported a positively changed view of life, known as posttraumatic growth (Linley & Joseph, 2004). Posttraumatic growth (PTG) describes the perceived positive changes in life view as a result of a personal recovery process. In other words, PTG results from more than simply the experience of the trauma (Calhoun & Tedeschi, 2006).

Research has illuminated aspects surrounding the phenomenon of PTG – including both functional growth with constructive and adaptive changes in survivors, and also more illusory aspects of growth such as self-deception, positive illusions, and avoidance (Hobfoll et al., 2007; Zoellner & Maercker, 2006). Several review articles about posttraumatic growth (Helgeson, Reynolds, & Tomich, 2006) show that many types of potentially traumatic events such as war and terrorism, loss, accidents, natural disasters, sexual abuse, and serious illnesses such as cancer, rheumatoid arthritis, heart attacks and AIDS, are strongly correlated with PTG. Some personality traits have also been shown to have a correlation with PTG. Tedeschi and Calhoun (2004) have found correlations with extraversion and openness to experience, as have Karanci et al. (2012) who reported correlations with conscientiousness and agreeableness. Women, younger people and those with more education tend to report a greater degree of PTG (Linley & Joseph, 2004; Tedeschi & Calhoun, 2004).

According to Tedeschi and Calhoun (2004), psychological distress, depression and general personal well-being are separate dimensions not directly related to PTG: ‘Posttraumatic growth is not the same as an increase in well-being or a decrease in distress’ (p. 13). Posttraumatic stress symptoms (PTSS) and PTG have shown positive (Solomon & Dekel, 2007), negative (Hall et al., 2008), and even curvilinear relationships (Kleim & Ehlers, 2009) with the highest levels of PTG corresponding to a medium-high level of PTSS. In a metaanalysis, Shakespeare-Finch and Lurie-Beck (2014) show significant linear and curvilinear correlations between PTG and PTSS, which were somewhat different depending on trauma type and age. The correlation between PTG and well-being has also been discussed (Zoellner & Maercker, 2006) both with regard to the length of time that has passed after the event, as well as the meaning of growth as a cognitive construction and change in action pattern (Hobfoll et al., 2007). The meaning of coping strategies, rumination, brooding, and core beliefs has also been studied in relation to PTG. After a natural disaster, both rumination and brooding were shown to have a mediating effect between perceived exposure and PTG (Garcia, Cova, Rincón, & Vázquez, 2015). Survivors’ re-evaluation of core beliefs was also related to PTG (Taku, Cann, Tedeschi, & Calhoun, 2015).

Tedeschi and Calhoun (1996) developed an inventory to assess posttraumatic growth (PTGI), which, according to Helgeson et al. (2006) is one of the most frequently applied instruments for this purpose. The inventory has been designed to assess various aspects of an individual’s perceived changed view of life, and as a result of factor analyses, it has been categorized into five general factors (relations to others, ability to see new opportunities, personal strength, spiritual change, and appreciation for life). Although these factors have been verified in several studies (Taku, Cann, Calhoun, & Tedeschi, 2008), using the inventory’s total sum is recommended (Osei-Bonsu, Weaver, Eisen, & Vander Wal, 2012). PTGI has also been translated into different languages and used in various cultures and countries where cultural-specific approaches towards potentially traumatic events needed to be considered (Splevins, Cohen, Bowley, & Joseph, 2010). The inventory’s reliability and validity have been proven in many studies (Shakespeare-Finch, Martinek, Tedeschi, & Calhoun, 2013).

Researchers have noted that although trauma survivors may experience positive change after a potentially traumatic event, they may also experience negative changes in life (Baker, Kelly, Calhoun, Cann, & Tedeschi, 2008; Barrington & Shakespeare-Finch, 2013), which could also constitute part of an adaptive process.

In order to better assess negative changes in life view (PTD) after a potentially traumatic event, Baker et al. (2008), added a negatively formulated question for each question in the original inventory. Their intent was to ‘… create a measure that would allow respondents to report a depreciation in the same domains in which they report growth on the PTGI.’ (Baker et al., 2008, p. 455). The PTG and PTD scales are statistically independent according to Baker et al. (2008).

A number of studies done after natural disasters have made use of PTG, but only a few have examined both PTG together with PTD (Baker et al., 2008; Barrington & Shakespeare-Finch, 2013; Cann, Calhoun, Tedeschi, & Solomon, 2010). The relation between PTG and PTD is of interest to study, as is the importance of different types of exposure for reported PTG and PTD, particularly in a longitudinal perspective. Our study addresses disaster survivors’ self-reported positively and negatively changed life views six years after a natural disaster – the 2004 tsunami in Southeast Asia.

## 2. Purpose

The purpose was to examine whether the type of exposure that survivors experienced during a natural disaster was of importance for their self-assessments of PTG and PTD six years after the disaster (T2), with controls for background variables. Additionally, the study examines the associations between general psychological distress and PTSS at T1 to PTG and PTD at T2, respectively. An additional purpose was to examine whether psychological distress or PTSS at T1 could have a mediating effect on PTG or PTD at T2 with gender, age and exposure as indirect and direct effects.

## 3. Material and method

A questionnaire was mailed on two occasions, 14 months (T1) and six years (T2) after the 2004 tsunami disaster. This study was conducted in collaboration with the National Centre for Disaster Psychiatry, Uppsala University and the Department of Medical Epidemiology and Biostatistics, Karolinska Institutet, Stockholm. The study has been reviewed by a regional research ethics committee in Stockholm.

### 3.1. Participants

In the weeks following the 2004 tsunami disaster in Southeast Asia, the police registered Swedes returning home. Based on their records, a questionnaire was sent to 4283 eligible persons 16 years and older who lived in Stockholm county at T1. After one reminder, a total of 1939 persons (45%) responded to the questionnaire. Of these, 1505 had been in the affected area when the tsunami swept in, thus excluding 434 respondents who had been in other places in Southeast Asia. The group of non-respondents contained significantly more younger people χ^2^ (4, *n *= 4276) = 132.29, *p *< 0.001 (seven did not answer the question) and men (61%) than women (48%) χ^2^ (1, *n *= 4283) = 77.91, *p *< 0.001. The 1505 people who had been in the disaster-stricken areas and who responded to the questionnaire at T1 were sent a new questionnaire six years after the disaster (T2). Of these, 848 people (56%) responded at T2, thus forming the project’s study group.

### 3.2. Measures

The questionnaire at T1 contained questions to assess gender, age, educational level (elementary school/high school/university), living situation (living alone/cohabitating), full-time work before the tsunami (yes/no) and whether respondents had been accompanied by children (yes/no). Four different types of exposure experienced by survivors have been assessed using four questions with a yes/no response: presence on the beach (including in the water) when the wave hit, experience of life threat, severe physical injury, loss of a significant person. Eight exposure categories were created based on survivors’ responses. The most exposed category was comprised of those who answered ‘yes’ to all four questions. Some exposure categories were created from a ‘yes’ response to one of the questions, while others were created from ‘yes’ responses to a combination of several questions. ‘No’ responses to all four questions constituted the category of least exposure, although survivors in this category were still somewhat exposed since they were present in the disaster-stricken area. The categories are presented in Table 2. This procedure to create exposure categories has been reported earlier (Wahlström, Michélsen, Schulman, & Backheden, 2008).

**Table 1.** Respondent characteristics, at T1 (%), *n *= 848.

	%
Gender:	
Women	62
Men	38
Age:	
16–24	10
25–34	18
35–44	22
45–54	28
≥55	22
Education:	
University	50
High school	38
Elementary school	12
Cohabitating	76
Full-time work before	
the tsunami	66
Accompanied by children	34

**Table 2.** Posttraumatic growth (PTG) and posttraumatic depreciation (PTD) at T2 according to single exposure and combinations of exposure, mean and standard deviation (SD), *n *= 848.

		PTG		PTD	
Exposure group	*n*	Mean SD		Mean SD	
Tsunami area only	289	22.61	22.69	6.97	10.03
Bereavement	27	36.08	24.81	8.00	9.14
Presence on beach	114	23.80	23.78	9.38	14.72
Life threat	149	36.71	23.62	14.12	16.80
Life threat and beach	149	37.17	23.44	13.51	14.95
Life threat, beach, and severe injury	28	38.19	22.02	15.35	19.67
Life threat, beach and bereavement	68	45.08	24.54	19.32	17.30
Life threat, beach, bereavement, and severe injury	24	42.64	23.62	19.14	15.08
Total	848	31.03	24.63	11.32	14.57

At T1 the 12-item General Health Questionnaire (GHQ-12) was used to identify general psychological distress rated over the past few weeks (Goldberg et al., 1997), a scale that is often used in population and trauma studies (Connor, Foa, & Davidson, 2006). Each question has four responses, 0–3. The higher the score, the more distressed the respondent. Responses were dichotomized in accordance with the designers’ instructions (Goldberg & Williams, 1988), whereby ratings of 0 or 1 are coded as ‘0’ and ratings of 2 or 3 as ‘1’, giving a range of 0–12. The Cronbach’s alpha was 0.92. The Impact of Event Scale Revised (IES-R) consisting of 22 questions was used to assess PTSS (Weiss, 2004). The degree of distress during the last week in response to a stressor is rated for each item on a 5-point scale, 0 = not at all to 4 = extremely, giving a range of 0 = 88. The stressor in this study was the 2004 tsunami. The Cronbach’s alpha was 0.95.

At T2, the Posttraumatic Growth Inventory (Tedeschi & Calhoun, 1996) was used, consisting of 21 questions containing various expressions for a positively changed view of life, where each question is answered on a scale 0–5, (0 = not having changed as a result of the disaster; 5 = having changed to a great extent). A total score for PTG was 0–105. Posttraumatic depreciation was included with 21 questions concerning different types of negative change in life after the disaster (Baker et al., 2008). These questions are negatively formulated in relation to each PTG question and are answered on a corresponding scale of 0–5. All 42 questions were first translated to Swedish, and then re-translated into English by independent professional translators, and then re-examined by experts in the area of trauma psychology (Guillemin, Bombardier, & Beaton, 1993). In contrast to Baker et al. (2008), who present the 42 questions in pairs, we randomly mixed the questions concerning growth and depreciation. The results of the 42 questions were then assigned to the two scales, PTG and PTD. The Cronbach’s alpha for PTG was 0.96 and for PTD was 0.94.

### 3.3. Data analysis

Pearson’s correlation was used for the relations between GHQ-12, IES-R, PTG and PTD. For non-response analyses we used log linear models for repeated measures. In order to investigate whether PTG and PTD reflected different aspects of a changed view of life, we used an explorative factor analysis  with the rotation method: promax. Linear regression models were conducted with PTG and PTD as outcome variables, with controls for gender, age, education, living situation, full-time work before the tsunami, accompanied by children, and type of exposure during the disaster. The distribution in the outcome variables was skewed, which is why data were log transformed for the regression analyses. The outcome of the analyses was anti-log transformed, with the estimate given in the form of a geometric mean ratio. The regression analyses were controlled for normal distribution of residuals, linearity, homogenous variances and outliers. Four pathway analyses have been conducted – two with PTG and two with PTD as outcome variables at T2. One analysis with PTG had GHQ-12 at T1 as a mediating variable, while the other had IES-R at T1 as a mediating variable. Corresponding analyses were done with PTD as an outcome variable. To analyse both indirect and direct effects, the three categorized variables gender, age and exposure were included in all four analyses. Bayesian linear regression and pathway analysis based on the Markov chain Monte Carlo method were used (Li, Schneider, & Bennett, 2007). All analyses were done with 30,000 iterations with controls for autocorrelation and tracing. Stability has been controlled for using Gelman–Rubin statistics. Before performing the pathway analysis, we examined the direction and strength of the correlations between variables with multiple regression analysis. SAS 9.1.3 software (SAS Inst. Inc., Cary, NC, USA) was used for statistical analyses, except for the pathway analyses which used WinBUGS version 1.4.3. (WinBUGS 1996–2007, Imperial College School of Medicine at St Mary’s, London, UK).

#### 3.3.1. Non-response analysis between the first and second measurements

We used log linear models to compare background variables and exposure at T1 for both respondents and non-respondents at T2. The interactions between the variables gender, age and education in relation to whether or not a respondent answered at T2 were significant. At T2, proportionally fewer men (*p *< 0.001), proportionally fewer in the youngest age group (*p *< 0.01), and proportionally fewer with only elementary school (*p *< 0.05) answered. For other background variables as well as type of exposure, the interactions regarding response or non-response at T2 were not significant. Nor were the interactions significant for the variables GHQ-12 and IES-R at T1 in relation to response or non-response at T2.

## 4. Results

The proportion of women in the study group was somewhat greater than the proportion of men, and the age group 45–54 years was somewhat larger than other age groups (Table 1). The level of education was high (50% had university or college education), 76% were cohabitating and 66% had full-time work before the tsunami.

**Figure 1.**
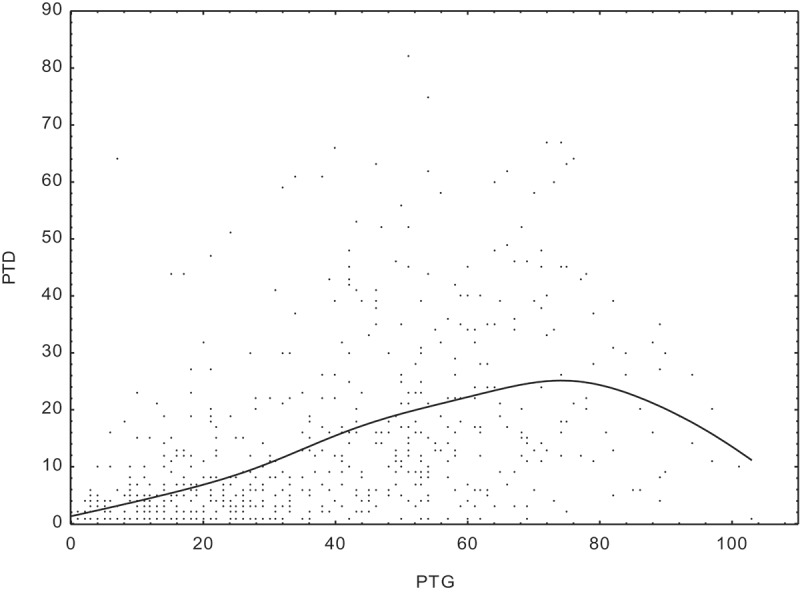
The association between the total score for posttraumatic growth (PTG) and posttraumatic depreciation (PTD).

The total PTG score was considerably higher than the total PTD score (mean for PTG = 31.0 and for PTD = 11.3). Both the PTG and PTD scores were higher for those who experienced combinations of two or more types of exposure (Table 2). The correlation between PTG and PTD was curvilinear (Figure 1), and in the interval 0–75 of PTG *r *= 0.57, while in the interval 76–105 *r *= −0.36. An explorative factor analysis, designed for two factors, showed that all PTG questions had higher factor loading in one factor while all PTD questions had higher factor loading in the other, indicating that PTG and PTD partly reflected different aspects of a changed view of life.

As a part of the study’s purpose to examine the correlations between GHQ-12 and IES-R at T1, and PTG and PTD at T2, the results showed correlations between r = 0.29 and r = 0.54, with higher correlations for IES-R (Table 3).

**Table 3.** Means, standard deviations (SD), Cronbach’s α, and correlations between General Health Questionnaire (GHQ-12), Impact of Event Scale Revised (IES-R) at T1 and posttraumatic growth (PTG), and posttraumatic depreciation (PTD) at T2.

	Mean	SD	Cronbach’s α	IES-R	PTG	PTD
GHQ-12	2.11	3.43	0.92	0.60	0.29	0.43
IES-R	21.84	18.28	0.95		0.47	0.54
PTG	30.98	24.63	0.96			0.55*
PTD	11.30	14.56	0.94			

All correlations are significant for *p* < 0.05

*The correlation between PTG and PTD is curvilinear.

The importance of different types of exposure in relation to PTG and PTD at T2, respectively, was studied using linear regressions. In the analysis with PTG as an outcome variable and with control for background variables and type of exposure, there emerged a significant geometric mean ratio for a high degree of PTG for the exposure groups that had experienced life threat, bereavement or a combination of exposures (geometric mean ratio between 1.56 and 2.02), compared to the group that had only been in the disaster-stricken area. Being exposed to several types of exposures resulted in higher geometric mean ratio. The age groups 35–44 and 45–54 had a significantly higher geometric mean ratio compared to older people, and the education level ‘high school’ also had higher scores (Table 4). Corresponding linear regression analysis with PTD as an outcome variable showed that a high degree of PTD was associated with type of exposure, age and education level. Exposure groups who experienced threat to life and combinations of the other types of exposures had significantly higher geometric mean ratios (2.18–3.93) compared to the group that had only been in the area, with the highest geometric mean ratio for the group with all four types of exposures. Age group 34–44 and age group 45–54 had higher geometric mean ratios compared to older groups, just as elementary and high school education had significantly higher geometric mean ratios compared to university educated (Table 4).

**Table 4.** Geometric mean ratio for reporting posttraumatic growth (PTG) and posttraumatic depreciation (PTD) at T2 according to different types of exposure, and co-variates at T1.

	PTG		PTD	
	Geometric mean ratio	95% Cl	Geometric mean ratio	95% Cl
Gender:				
Women	1.0		1.0	
Men	0.83	0.71–0.95	0.86	0.71–1.04
Age:				
>55	1.0		1.0	
45–54	1.29*	1.04–1.58	1.36*	1.03–1.80
35–44	1.36**	1.07–1.71	1.53**	1.11–2.10
25–34	1.14	0.91–1.43	1.23	0.91–1.66
16–24	0.97	0.75–1.27	0.93	0.65–1.33
Education:				
University	1.0		1.0	
High school	1.24**	1.07-1.44	1.39**	1.14–1.70
Elementary school	1.14	0.90-1.44	1.86***	1.36–2.53
Living situation:				
Single	1.0		1.0	
Cohabitating	1.03	0.88-1.22	1.14	0.91–1.42
Full-time work before the tsunami:				
No	1.0		1.0	
Yes	0.90	0.77–1.05	0.99	0.80–1.22
Accompanied by children:				
No	1.0		1.0	
Yes	1.08	0.92–1.28	1.06	0.85–1.32
Exposure group:				
Tsunami area only	1.0		1.0	
Bereavement	1.70**	1.15–2.51	1.26	0.76–2.10
Presence on beach	1.00	0.80–1.24	1.19	0.89–1.59
Life threat	1.56***	1.28–1.90	2.18***	1.67–2.85
Life threat and beach	1.76***	1.45–2.14	2.18***	1.67-2.83
Life threat, beach, and severe injury	1.92***	1.32–2.79	2.64***	1.56-4.46
Life threat, beach, and bereavement	2.02***	1.56–2.62	3.35***	2.36-4.76
Life threat, beach, bereavement, severe injury	1.89**	1.26–2.83	3.93***	2.24–6.88

*p < 0.05, **p < 0.01, ***p < 0.001.

The study’s third purpose was investigated using a pathway analyses that showed that both GHQ-12 and IES-R at T1 were significant mediator variables for the outcome in both PTG and PTD at T2 (Table 5). It also emerged that gender, age and type of exposure had significant indirect effects on PTG with GHQ-12 as mediator variable. With IES-R included as a mediator variable gender and type of exposure had significant indirect effects on PTG. Corresponding analyses with PTD as an outcome and GHQ-12 as mediator variable showed that gender, age and type of exposure had significant indirect effects. With IES-R as mediator variable gender and type of exposure had significant indirect effects.

**Table 5.** Bayesian pathway analysis with general psychological distress (GHQ-12) and posttraumatic stress symptoms (IES-R), 14 months post-disaster (T1), as mediator variables for the outcome variables, posttraumatic growth (PTG) and posttraumatic depreciation (PTD), six years post-disaster (T2). To estimate direct and indirect effects, the three categorized variables gender, age and exposure were used in the statistical models. Significant indirect effect coefficients with 95% confidence interval are presented.

	PTG		PTD	
Mediator variable	b*	CI 95 %	b*	CI 95 %
GHQ-12	1.65	1.17-2.13	1.66	1.39-1.92
Indirect effect				
GHQ-12	effect coefficient		effect coefficient	
Gender/men	1.05	0.25-1.96	1.04	0.26-1.87
Age/25-34	1.66	0.43-3.08	1.64	0.43-2.92
Age/16-24	2.30	0.82-4.00	2.27	0.83-3.77
Exposure				
Life threat, beach, and severe injury	2.85	0.67-5.32	2.82	0.67-5.05
Life threat, beach, and bereavement	3.77	2.06-5.76	3.72	2.18-5.39
Life threat, beach, bereavement, severe injury	6.01	3.31-9.13	5.94	3.48-8.59
Mediator variable	b*	CI 95 %	b*	CI 95 %
IES-R	0.54	0.45-0.64	0.42	0.37-0.48
Indirect effect IES-R	effect coefficient		effect coefficient	
Gender/men	3.72	2.32-5.24	2.81	1.74-3.95
Exposure				
Life threat	6.17	4.13-8.38	4.74	3.21-6.41
Life threat and beach	5.71	3.69-7.93	4.57	3.02-6.20
Life threat, beach and severe injury	8.25	4.60-12.19	6.82	3.92-9.85
Life threat, beach, and bereavement	11.77	8.72-15.09	9.16	6.94-11.52
Life threat, beach, bereavement, severe injury	11.86	7.51-16.60	9.30	6.01-12.80

Results from the complete pathway analyses with indirect, direct and mediating effects have been presented as supplementary material for the article.

* regression coefficient for mediator variables.

## 5. Discussion

In this study, where both posttraumatic growth (PTG) and posttraumatic depreciation (PTD) were studied in relation to the same potentially traumatic event – a natural disaster – one of the main results was that the type of exposure, and especially the combination of exposures experienced by survivors during the disaster had strong associations to both PTG and PTD six years after the disaster. Survivors who experienced a combination of exposures reported significantly higher values in both PTG and PTD compared to those who had only been in the disaster-stricken area. Positive correlations emerged between IES-R (T1) and PTG and PTD respectively (T2), which was higher than the corresponding correlation to GHQ-12 (T1). Pathway analyses showed that psychological distress (GHQ-12) and posttraumatic stress symptoms (IES-R) 14 months post-disaster (T1), were significant mediator variables for both PTG and PTD six years post-disaster (T2).

The respondents reported considerably more positive than negative changes in life view. Among those who were in the upper quartile for PTG were 114 people (13% of the study group) who could also be found in the upper quartile for PTD – a result in line with what Baker et al. (2008) found among undergraduate students. Cann et al. (2010) and Barrington and Shakespeare-Finch (2013) have shown low correlations (*r *= 0.10) between the two scales. However, we found a curvilinear association with a strong positive correlation up to a total score of 75 in PTG, from which a weak negative correlation emerged (Figure 1). A completed factor analysis showed that PTG and PTD questions broke down into separate factors. These results indicate that the two scales measure somewhat different aspects, and that PTG and PTD do not work as opposing questions on the same continuum, which concurs with Barrington and Shakespeare-Finch (2013). The two scales provide a broader image of the respondents’ changed view of life, giving them both a place in studies of disaster survivors.

There is reason to note the length of time between the disaster and the point at which PTG and PTD data were collected. Our study group was comprised of survivors of the same disaster who responded to a questionnaire six years afterwards. In previous studies evaluating both PTG and PTD, respondents reported various types of potentially traumatic events that had occurred between 0 and 36 months prior to the study (average value was 15 months) (Cann et al., 2010) and maximum of five years (average was 26 months) (Barrington & Shakespeare-Finch, 2013), respectively, before they responded to the questionnaire.

Positive correlations between PTSS and PTG, meaning that those presenting more posttraumatic symptoms also reported more posttraumatic growth, have been reported in many studies (Zoellner & Maercker, 2006). The same correlations emerged in this study between IES-R (T1) and PTG (T2). Both linear and curvilinear correlations between PTSS and PTG have previously been described (Kleim & Ehlers, 2009; Shakespeare-Finch & Lurie-Beck, 2014). Lowe, Manove, and Rhodes (2013) found a positive correlation in which higher PTSS both one and three years after a disaster resulted in higher PTG three years after the disaster. To our knowledge, a correlation between PTSS and PTD has not been reported. This study showed a significant correlation between IES-R and PTD, which is to say that survivors with high PTSS at T1 reported that they experienced strong negative changes in life six years post disaster, at T2.

In previous studies of the correlation between general psychological distress and PTG, the results are not completely unanimous. Several studies have not shown any correlation while others have shown weak correlations (Hobfoll et al., 2007; Zoellner & Maercker, 2006). Our study showed a weakly positive correlation between GHQ-12 (T1) and PTG (T2), and a somewhat stronger correlation with PTD (T2). The negatively formulated questions may possibly reflect additional aspects of psychological distress, something suggested by Barrington and Shakespeare-Finch (2013).

In previous studies, women, younger people and those with higher education have estimated PTG to be higher (Linley & Joseph, 2004; Solomon & Dekel, 2007). This study showed a somewhat different result. In linear regression models gender was not significant either in relation to PTG or PTD, and middle-aged people and those with high school educations had both higher PTG and PTD – a result that is partly in line with Jin, Xu, Liu, and Liu (2014).

PTG has been studied after natural disasters to a lesser degree than other types of serious events such as illness, war, sexual abuse and violence (Helgeson et al., 2006). The results consistently show a positive correlation between exposure and high values for PTG. Our study looked at tourists who were struck by the 2004 tsunami disaster while visiting Southeast Asia. Residents of the afflicted countries have not been included in the study. The importance of exposure type was striking, with a falling geometric mean ratio in both PTG and PTD for those who had been affected by several types of exposure, compared to those who reported few types. Similar results regarding the importance of exposure during a natural disaster and subsequently reported PTG have been shown by Jin et al. (2014), who studied those affected and unaffected one year after an earthquake. Marshall, Frazier, Frankfurt, & Kuijer (2014) who, in a longitudinal design investigated both PTG and PTD among survivors who had experienced two consecutive earthquakes, found no correlation between the extent of exposure and PTG/PTD one year after the second earthquake. One explanation for this outcome may be that the extent of the exposure in Marshall’s et al. (2014) study was much less compared to our study, since none of the survivors reported any loss of family members or neighbours. What is more, Marshall et al. (2014) estimated positively and negatively changed views of life with values on the same continuum, in contrast to both this study and recommendations from Barrington & Shakespeare-Finch (2013).

The results of pathway analyses gave new perspectives on associations between exposure, PTSS and general psychological distress and the studied outcome variables. This study shows that different types of exposure during the disaster had both a direct and an indirect effect on PTG and PTD six years after the disaster. In addition, both PTSS and general psychological distress 14 months after the disaster (T1) had a mediating effect on both PTG and PTD six years after the disaster (T2). Other studies have shown that rumination processes (Garcia et al., 2015) had a mediating effect on PTG, while this study showed that both previous PTSS and general psychological distress (T1) had a mediating effect on both a positively and negatively changed life view six years after the disaster (T2). The fact that high values for PTSS were important for high values in PTG several years later is in line with Lowe et al. (2013). In our study it was also clear that general psychological distress had a mediating effect on subsequent PTG. These results support future theories that personal recovery processes may occur in people who have experienced a potentially traumatic event and that this process is mediated by previously expressed PTSS and/or general psychological distress. That personal recovery processes after a disaster may also include perceived negative changes in life view (Baker et al., 2008; Barrington & Shakespeare-Finch, 2013) was also supported in our study. This underscores the importance of studying how disaster survivors report both positive and negative changes in life view after a potentially traumatic event.

### 5.1. Strengths and limitations

The answer frequency of 45% at T1 constitutes a weakness in this study, but it is similar to other European studies of natural disasters (Hussain, Weisaeth, & Heir, 2009). In a previous study, when weighting to compensate for nonresponse, the outcome in GHQ-12 and IES-R at T1 did not change in any significant way (Wahlström et al., 2008). In this study, we imputed the missing data regarding the outcome variables PTG and PTD (T2). After weighing together results of five imputations for PTG and PTD, respectively, no changes emerged regarding significant/non-significant variables that were included in the presented linear regressions with PTG and PTD as outcome variables. The study lacks information regarding life events and psychological distress before the tsunami. However, the strengths are the longitudinal design and a large group of survivors who answered the questionnaires at 14 months (T1) and six years after the disaster (T2). Socioeconomic data (T1) showed that the survivors had a generally high level of education, a high proportion of full-time work, a high proportion of cohabitation, and that they travelled with children on vacation in Southeast Asia. These factors indicate that one could expect a low level of psychological distress before the disaster in the study group. The group was relatively ethnically homogenous. Although this limits generalizability, the group was not affected by other religious or cultural differences, which may be of particular importance for interpreting PTG (Splevins et al., 2010) and PTD.

Regarding exposure during the disaster, the data were retrospective, (T1), with a risk for recall bias, but there was no selection bias regarding the background variables in relation to type of exposure. Self-reported PTG and PTD gave no differential information about whether the reported changes were of a more illusory type or reflected direct changes in behaviour.

During natural disasters many people lose their homes, their employment conditions, their social network, and often the physical infrastructure of society. None of these consequences affected the study group, who were able to fly home a few days after the disaster (Sattler, Assanangkornchai, Moller, Kesavatana-Dohrs, & Graham, 2014). This limits the generalizability of the study while also allowing us to examine and compare different types of exposure in a much more isolated way.

All study participants had in some way been exposed to the disaster, even those who, according to the exposure categories, constituted the comparison group in the regression analyses. This means that the geometric mean ratios that were calculated should not be seen as overestimates of reported risks.

Continued research of both the PTG and PTD scales, particularly in relation to social support, quality of life, and meaning in life is necessary for many reasons, as are further studies of the three factors in IES-R in relation to PTG and PTD (Cann et al., 2010). Longitudinal studies are needed to explore how changes in PTSS and PTG/PTD are related over time (Lowe et al., 2013). There is also a need to develop the theoretical models of posttraumatic change that take PTD into account.

## 6. Conclusions

PTG and PTD worked well as separate scales of perceived changed view of life after a natural disaster, and they show that growth and depreciation can coexist. Both PTG and PTD have proven to be related to type of exposure. PTSS and general psychological distress have a mediating effect on subsequently reported PTG and PTD. Studying disaster survivors’ changed view of life after a potentially traumatic event, both in terms of posttraumatic growth and posttraumatic depreciation, provided a broader understanding of the complexity of the reactions and recovery process of the survivors.

## Supplementary Material

Supplementary material

## Highlights of the article

Self-reported posttraumatic growth and posttraumatic depreciation reveal different pictures of the survivors’ changed view of life.

Different types of exposures showed clear associations to survivors’ reports of both PTG and PTD six years post disaster.

PTSS and psychological distress one year after a natural disaster were positively correlated with both PTG and PTD six years post disaster.

