# Supplementary material for: Posttraumatic growth and depreciation six years after the 2004 tsunami
Source: Eur J Psychotraumatol. 2017 Mar 24;8(1):1302691. doi: 10.1080/20008198.2017.1302691 (PMC5399995; doi:10.1080/20008198.2017.1302691)
Supplement: Supplementary material [file zept_a_1302691_sm0048.docx]

**Supplementary material for the article.**

titled

Posttraumatic growth and depreciation six years after the 2004 Tsunami

*Table 5a.* Bayesian path way analysis with general psychological distress (GHQ 12), 14 months post disaster (T1), as mediator variables for the outcome variables, posttraumatic growth (PTG) and posttraumatic depreciation (PTD), 6 years post disaster (T2). For the estimation of indirect and direct effects the three categorized variables gender, age and exposure were used in the statistical models.

|  | PTG |  | |  |  | | PTD | | | |  |
| --- | --- | --- | --- | --- | --- | --- | --- | --- | --- | --- | --- |
| Indirect effect  Gender  women  men | effectcoefficient  1.05 | CI 95 %  0.25-1.96 | |  |  | | effectcoefficient  1.04 | | | | CI 95 %  0.26-1.87 |
|  |  |  | |  |  | | | | |  |  |
| Age |  |  | |  |  | | | | |  |  |
| >=55  45-54  35-44  25-34  16-24 | 1.02  1.01  1.66  2.30 | -0.05-2.21  -0.14-2.28  0.43-3.08  0.82-4.00 | |  |  | | 1.03  1.00  1.64  2.27 | | | | -0.04-2.14  -0.14-2.19  0.43-2.92  0.83-3.77 |
| Exposure | |  | |  |  | |  | | | |  |
| Tsunami area only  Bereavement  Presence on beach  Life threat  Life threat and beach  Life threat, beach, and severe injury  Life threat, beach, and bereavement  Life threat, beach, bereavement, severe injury | 1.20  0.39  0.92  1.06  2.85  3.77  6.01 | -1.00-3.54  -0.84-1.66  -0.19-2.16  -0.05-2.29  0.67-5.32  2.06-5.76  3.31-9.13 | |  |  | | 1.18  0.41  0.91  1.05  2.82  3.72  5.94 | | | | -0.99-3.43  -0.80-1.63  -0.19-2.07  -0.05-2.19  0.67-5.05  2.18-5.39  3.48-8.59 |
|  |  |  | |  |  | | |  | | |  |
|  |  |  | |  |  | | |  | | |  |
| Mediating effect b* | | CI 95 % | |  | | | | b* | | | CI 95 % |
|  |  |  | |  | | | |  | | |  |
| GHQ-12 | 1.65 | 1.17-2.13 | |  | | | | 1.66 | | | 1.38-1.92 |
|  |  |  | |  | | | | | |  |  |
| Direct effect | |  | |  | | | | | |  |  |
|  |  |  | |  | | | | | |  |  |
| Gender  women  men | 4.26 | 0.98-7.51 | |  | | | | | -0.18 | | -2.01-1.64 |
|  |  |  | |  | | | | |  | |  |
| Age |  |  | |  | |  | | |  | |  |
| >=55  45-54  35-44  25-34  16-24 | 6.34  8.16  2.70  2.77 | 1.84-10.83  3.44-12.90  -2.33-7.77  -3.09-8.66 | | | |  | | | -0.45  0.27  -2.42  -2.91 | | -2.97-2.07  -2.38-2.96  -5.27-0.42  -6.25-0.42 |
| Exposure | |  | |  | |  | | |  | |  |
| Tsunami area only  Bereavement  Presence on beach  Life threat  Life threat and beach  Life threat, beach, and severe injury  Life threat, beach, and bereavement  Life threat, beach, bereavement, severe injury | 11.24  1.00  12.08  12.64  12.40  16.83  11.58 | 2.00-20.29  -4.01-5.94  7.54-16.76  8.03-17.23  3.55-21.27  10.69-23.04  1.47-21.75 | | | |  | | | -0.28  2.01  6.19  5.05  5.00  7.41  5.29 | | -5.39-4.75  -0.78-4.79  3.63-8.79  2.47-7.63  0.03-9.96  3.99-10.91  -0.34-10.98 |
|  |  | |  | | |  | | | |  |  |
|  |  | |  |  | |  | | | |  |  |

*regression coefficient for mediator variables

*Table 5b.* Bayesian path way analysis with posttraumatic stress symptoms (IES-R), 14 months post disaster (T1), as mediator variable for the outcome variables, posttraumatic growth (PTG) and posttraumatic depreciation (PTD), 6 years post disaster (T2). ). For the estimation of indirect and direct effects the three categorized variables gender, age and exposure were used in the statistical models.

|  |  |  |  |  | |  |  |
| --- | --- | --- | --- | --- | --- | --- | --- |
|  | PTG |  |  |  | | PTD |  |
| Indirect effect  Gender  women  men | effectcoefficient  3.72 | Cl 95 %  2.32-5.24 |  |  | | effectcoefficient  2.81 | Cl 95 %    1.74-3.95 |
| Age |  |  |  | | |  |  |
| >=55  45-54  35-44  25-34  16-24 | -1.22  -1.12  -0.92  1.35 | -3.07-0.56  -3.05-0.79  -2.97-1.10  -1.02-3.77 |  |  | | -1.05  -0.82  -0.99  1.01 | -2.49-0.39  -2.35-0.70  -2.61-0.61  -0.88-2.93 |
|  |  |  |  |  | |  |  |
| Exposure | |  |  |  | |  |  |
| Tsunami area only  Bereavement  Presence on beach  Life threat  Life threat and beach  Life threat, beach, and severe injury  Life threat, beach, and bereavement  Life threat, beach, bereavement, severe injury | 3.32  0.71  6.17  5.71  8.25  11.77  11.86 | -0.34-7.12  -1.30-2.78  4.13-8.38  3.69-7.93  4.60-12.19  8.72-15.09  7.51-16.60 |  |  | | 2.49  0.99  4.74  4.57  6.82  9.16  9.30 | -0.33-5.36  -0.60-2.62  3.21-6.41  3.02-6.20  3.92-9.85  6.94-11.52  6.01-12.80 |
|  |  |  |  |  | |  |  |
| Mediating effect b* | | CI 95 % |  | | | b* | CI 95 % |
|  |  |  |  | | |  |  |
| IES-R | 0.54 | 0.45-0.64 |  | | | 0.42 | 0.37-0.48 |
|  |  |  |  | | |  |  |
| Direct effect | |  |  | | |  |  |
|  |  |  |  | | |  |  |
| Gender  women  men | 0.80 | -2.37-3.94 | | | | -2.21 | -4.10--0.34 |
|  |  |  |  | | |  |  |
| Age |  |  |  | |  |  |  |
| >=55  45-54  35-44  25-34  16-24 | 8.24  10.39  4.99  2.87 | 3.94-12.56  5.92-14.93  0.19-9.81  -2.77-8.46 | | |  | 1.72  1.81  -0.56  -1.50 | -0.84-4.29  -0.88-4.48  -3.41-2.28  -4.92-1.87 |
| Exposure | |  |  | |  |  |  |
| Tsunami area only  Bereavement  Presence on beach  Life threat  Life threat and beach  Life threat, beach, and severe injury  Life threat, beach, and bereavement  Life threat, beach, bereavement, severe injury | 7.56  0.84  7.15  8.52  7.37  8.09  8.77 | -1.04-16.20  -3.92-5.67  2.65-11.72  4.02-13.07  -1.02-15.87  1.84-14.34  -1.20-18.69 | | |  | -1.53  1.77  2.55  2.83  1.79  3.17  3.93 | -6.55-3.52  -1.07-4.61  -0.10-5.21  0.12-5.57  -3.38-6.95  -0.51-6.89  -1.83-9.71 |
|  |  |  | | |  |  |  |

*regression coefficient for mediator variables
